# Longitudinal evaluation, acceptability and long-term retention of knowledge on a horizontally integrated organic and functional systems course

**DOI:** 10.1007/s40037-015-0195-7

**Published:** 2015-07-31

**Authors:** Joana Almeida Palha, Armando Almeida, Jorge Correia-Pinto, Manuel João Costa, Maria Amélia Ferreira, Nuno Sousa

**Affiliations:** 1 Life and Health Sciences Research Institute (ICVS), School of Health Sciences, University of Minho, Campus Gualtar, 4710-057 Braga, Portugal; 2 Center for Medical Education, Faculty of Medicine, University of Porto, Porto, Portugal; 3 Life and Health Sciences Research Institute (ICVS), School of Health Sciences, ICVS/3B’s—PT Government Associate Laboratory, Braga/Guimarães, and Clinical Academic Center-Braga (2CA), University of Minho, Campus Gualtar, 4710-057 Braga, Portugal

**Keywords:** System-based, Curriculum, Medicine, Teaching

## Abstract

Undergraduate medical education is moving from traditional disciplinary basic science courses into more integrated curricula. Integration models based on organ systems originated in the 1950s, but few longitudinal studies have evaluated their effectiveness. This article outlines the development and implementation of the Organic and Functional Systems (OFS) courses at the University of Minho in Portugal, using evidence collected over 10 years. It describes the organization of content, student academic performance and acceptability of the courses, the evaluation of preparedness for future courses and the retention of knowledge on basic sciences. Students consistently rated the OFS courses highly. Physician tutors in subsequent clinical attachments considered that students were appropriately prepared. Performance in the International Foundations of Medicine examination of a self-selected sample of students revealed similar performances in basic science items after the last OFS course and 4 years later, at the moment of graduation. In conclusion, the organizational and pedagogical approaches of the OFS courses achieve high acceptability by students and result in positive outcomes in terms of preparedness for subsequent training and long-term retention of basic science knowledge.

## Introduction

Undergraduate medical curricula with discipline designs for the basic sciences are being replaced by curricula with units that integrate several disciplines [1–5]. One way to integrate the basic sciences is to combine multiple disciplinary perspectives into courses that address organ systems, providing clinical contextualization [4–9]. With the exception of problem-based learning [10, 11], there is little information on the effectiveness of such means of integration. Here, we describe the Organic and Functional Systems (OFS) courses that integrate anatomy, biochemistry, embryology, histology and physiology. We use data collected longitudinally for 10 years to address the following questions: (i) how do students perform and how do students appreciate OFS?; (ii) how do OFS courses prepare students for subsequent courses and clerkships?; and (iii) is there long-term retention of basic science knowledge?

## Methods

### Institutional context

OFS courses are part of the undergraduate medical programme of the School of Health Sciences, University of Minho, Braga, Portugal. The number of students admitted per course was initially 50 and is currently 120. There are three OFS courses (OFS1, OFS2 and OFS3) which take place in the first and second year of a six-year study plan.

### Description of the OFS courses and summative assessment

**Fig. 1:**
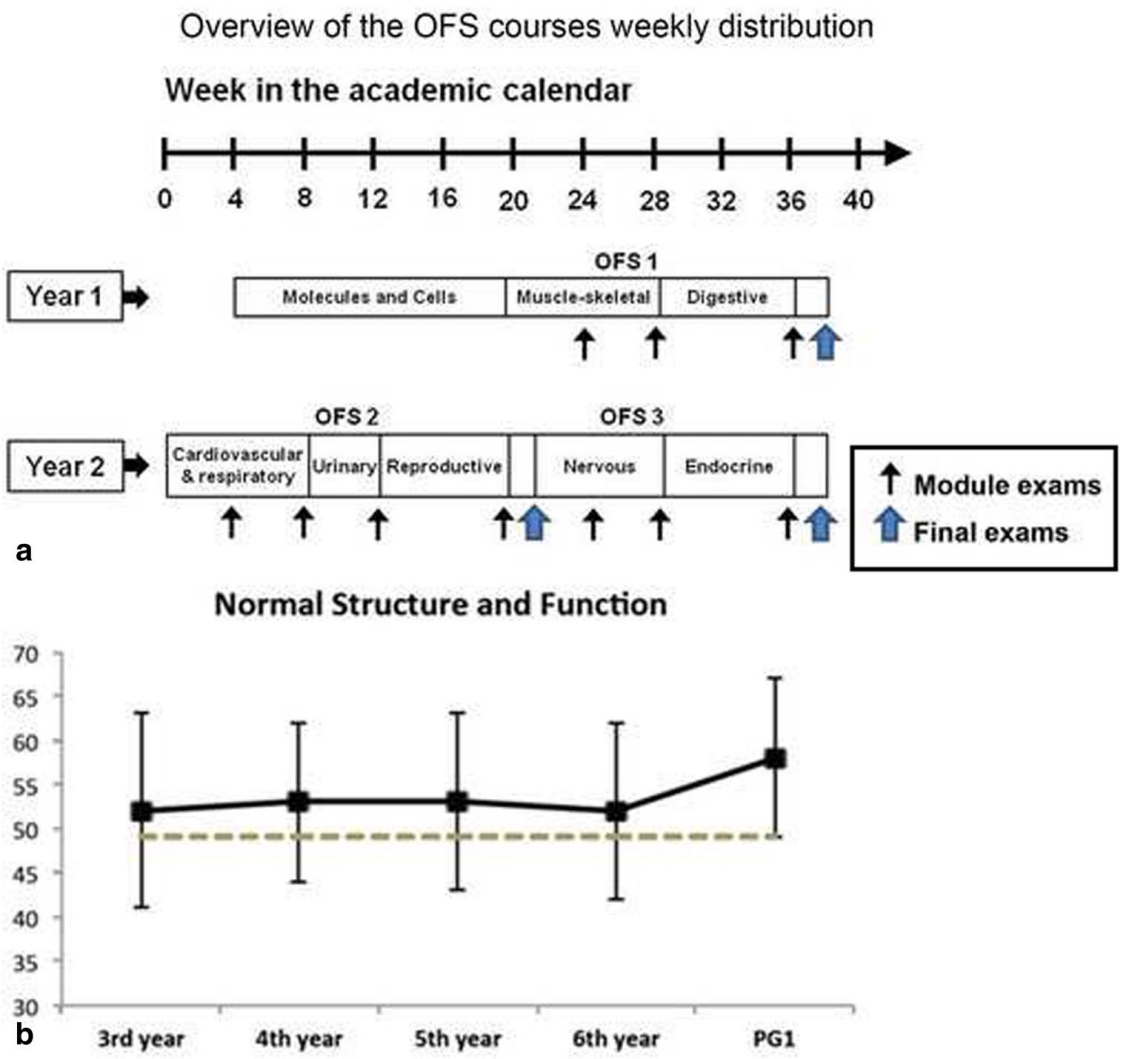
**a** Overview of OFS courses. Overview of the weekly distribution of OFS courses. Thin arrows represent module exams and large arrows represent integrated and clinical skills final exams. The course ‘Molecules and Cells’ precedes the first OFS. **b** Results for ‘Normal Structure and Function’ of the International Foundations of Medicine Exam. Results are in percent correct scores. Solid lines represent the performance of Minho’s students and dashed lines represent the performance of students of all medical schools who took the exam. Students taking the exam were in the third to sixth years of the 6-year medical course and new graduates (PG1)

The OFS courses present a morphofunctional perspective of each body system integrating anatomy, histology, embryology, physiology and biochemistry. Integration is accomplished by proposing learning objectives in the various disciplines that simultaneously tackle the same or complementary organ/function/mechanism. Each of the 3 OFS courses lasts for approximately 14 weeks, and is sub-divided into sequential modules (Fig. 1a).

Each module follows a pedagogical cycle with 5 phases including tutorials, supervised (ratio teacher/student from 1/8 to 1/45, depending on the activity) or self-directed learning activities, and examinations.

All students complete the same assessment programme, which includes high stake assessments: 1) written tests at the end of each module and one final case-based exam at the end of each OFS; 2) observational exams of practical and elementary clinical skills; 3) a cumulative mark for student behaviour in supervised activities. The Portuguese administrative regulations determine that final marks are numerical, on a scale of 0–20, with 10 being the pass/fail cut-off score.

### Population of the study

Records of student academic performance and student evaluations of teaching across a 10-year period (2001–10) were reviewed: 770 students, 66 % females, with a mean age at entry of 18.8 years (SD = 2.0).

### Acceptability of the programme

Acceptability by students was assessed by anonymous questionnaires delivered 2–4 weeks after the end of each OFS course. The questionnaires consisted of Likert-type statements (with 6 points ranging from ‘strongly disagree’ to ‘strongly agree’). The same questionnaires have been in use since the year 2005–2006, and for that reason the correspondent 5 cohorts were analyzed. The results were dichotomized; therefore, the percentages represented correspond to the sum of the positive answers ‘4, 5 and 6’.

### Analysis of long-term retention of knowledge

To measure student proficiency in basic and clinical sciences, their performance in the 2008 edition of the International Foundations of Medicine (IFOM) examinations (a multiple-choice assessment developed by a consortium of international medical schools led by the National Board of Medical Examiners) was analyzed. The blueprint was developed collaboratively and, in the 2008 edition, included 75 basic sciences (derived from the United States Medical Licensing Examination (USMLE) step 1 item bank) and 125 clinical items (derived from the USMLE step 2 item bank). Of these, 42 items regarded ‘Normal structure and function’ and, thus, of relevance to OFS courses. Items were administered in English— a second language for most students—in a low-stake examination. Participation in the examination was voluntary and offered without costs. Invitations were sent by email and 54 % (145 out of 266) voluntarily participated (33, 54, 75, 66 and 50 %, from third- to sixth-year students and recent graduates, respectively) [12]. Students within the whole range of academic performances in OFS courses volunteered to participate. All students took the same exam.

### Ethics

The use of academic performances for research was authorized by the Portuguese commission for data protection (CNPD: 10432/2011). Results from student ratings and the IFOM are anonymous.

## Results

### Academic performance

The OFS course marks for successful students were stable across the years with average marks of 12.5, 13.1 and 13.6, out of 20, for OFS1, OFS2 and OFS3, respectively.

With respect to the failure rates, there are two distinct periods: the initial 6 years, where the failure rate was of 0–9 %, and the last 4 cohorts in which it rose to 33 %. This increase in failure rate coincided with a doubling in student intake and is partially due to an increase in non-attendance (from 0 to 4 % in the initial 6 and up to 10 % in the last 4 cohorts).

### Acceptability of the programme

The ratings of the OFS courses were consistently high over the years. As to the ‘course evaluation’ questionnaire (available upon request) the lowest percentage of students who agree with the statement ‘Globally, I consider this curricular unit excellent’ was 70 %, with an average of 87 % across the years. There is one item with a consistently lower percentage of agreement, ‘The workload was appropriate considering the time programmed for learning’. Of note, the level of agreement increases from OFS1, to OSF2 and to OFS3. Student evaluation of the faculty (questionnaire available upon request) is extremely positive, with all items rated between 82 and 89 %.

### Preparedness for further training and retention of knowledge

The course evaluation questionnaire asks students about preparedness provided by previous courses. At the conclusion of the course, immediately after OFS3, ‘Biopathology and Introduction to Therapeutics’, 73–88 % of the students considered the previous training appropriate; a substantial part of this training was provided by OFS courses. Similarly, 90–94 % of the clinical tutors on the clerkships of the fourth and fifth years of the medical degree considered students’ skills appropriate at the start of their clinical clerkship.

The IFOM scores offer evidence of retention of knowledge on the normal structure and function of the body (Fig. 1b). The reliability of the IFOM exam was 0.90 [12]. When compared with the performance of students enrolled in the 3rd year (right after completing the basic science courses), it is of note that the basic science knowledge is retained with advancing years in the medical degree, when students are mostly in clinical training and even in those who had just graduated.

## Discussion

The first 10 years of experience suggest that the model has high acceptability by students and contributes to the long-term retention of basic sciences.

The academic performance of students in the 3 OFS courses was stable across the years and was within the internationally acceptable scores of performance.

The stability of scores likely reflects the general constancy of admission criteria. In the academic year 2007–08, the number of students entering the programme doubled and special entry regimes were decreed by Government. Coincidentally, there was a relevant increase (from 5 to approximately 30 %) in the failure rate of OFS courses. The reasons for such relatively high failure rates are difficult to identify as the academic performance of students is based on a combination of individual, circumstantial and institutional variables. Nevertheless, one can speculate that important elements resided in students admitted through the new regimes and coincidental changes in the application of university regulations to the medical degree. New university regulations discontinued a former prerogative that prevented students who failed OFS1 from registering in year 2 OFS. There were students unsuccessful in OFS1 in the previous year, who were also simultaneously following the year 2 OFS3 course for the first time, and the number of students failing for non-attendance increased in that year. While a detailed analysis of all these aspects deserves consideration, it should be noted that, altogether, the performance data indicate that students adapt to the model, since those who fail are mostly in OFS1. Also of relevance is the fact that over time, the failure rate has decreased significantly in the OFS2 and OFS3; currently it stands at around 6 % but remains high in OFS1.

Student evaluations of the courses and of the faculty were also stable over time and clearly demonstrate the high acceptability of the courses. The least appreciated items relate to the workload and to the assessment processes. It is important to note, however, that the appreciation in these items increased across the OFS, which may be ascribed to a greater adaptation of the students to the cognitive complexity of the OFS. Adaptation to an increasing workload and the perspective with which students face it is a common feature in educational settings. Of interest, on the yearly formal face-to-face encounter with students to reflect on the previous academic year, students usually relativize the workload on the previous courses in face of those they are going through at the moment. However, throughout the years, and in response to this concern, the weekly schedule has been adapted to allow for more specific periods of self-study.

In the IFOM exam students revealed long-term (up to postgraduate year 1) retention of knowledge of basic sciences. This is a positive indicator of the quality of the programme and suggests that the educational strategies implemented in OFS courses are successful. A note should be made about the higher performance of our students when taking the high stake national residency exam (which ranks them for the residencies programmes within the Portuguese National Health Service). This is a multiple choice test on internal medicine, but knowledge is expected to derive from the various fundamental disciplines, including the basic sciences. In this national residency selection exam, these very same cohorts of students ranked above those from all other Portuguese medical schools [13]. Additional studies are needed to determine whether the integrated teaching of basic disciplines within a clinical case-based approach fostered by the University of Minho medical degree contributes to retention of basic knowledge and on the student performances even in more clinical-related subjects.

We are aware that this study suffers from methodological limitations, the most important being the unavailability of data to demonstrate that all passing students meet the required standards in knowledge and skill proficiency. We do not have external examinations at the end of OFS courses to demonstrate competency or on the reliability and validity of the assessment programme as a whole. Indeed, the academic performance results originate from in-house assessments. In addition, since the courses have been integrated and system-based since the launching of the University of Minho medical degree, it was impossible to compare with previous discipline-based approaches with the same teaching staff and institutional setting. Such flaws are common in uncontrolled real world educational studies. As to the IFOM, we are aware that students self-selected to participate and that a direct association of student’s performance with the OFS experience cannot be established. We argue, however, that the overall coherency of data collected from different sources at different time points suggests that the OFS course fulfil their educational objectives.
